# Smartphone Video Imaging Combined with Machine Learning: A Cost-Effective Method for Authenticating Whey Protein Supplements

**DOI:** 10.3390/foods14071277

**Published:** 2025-04-05

**Authors:** Xuan Tang, Wenjiao Du, Weiran Song, Weilun Gu, Xiangzeng Kong

**Affiliations:** 1 School of Physical Education, Yunnan University, Kunming 650091, China; tangxuan@ynu.edu.cn (X.T.); duwenjiao_d@163.com (W.D.); 2 State Key Laboratory of Power System Operation and Control, Tsinghua University, Beijing 100084, China; 3 School of Future Technology, Haixia Institute of Science and Technology, Fujian Agriculture and Forestry University, Fuzhou 350002, China; xzkong@fafu.edu.cn; 4 School of Integrated Circuits, Jiangnan University, Wuxi 214122, China

**Keywords:** whey protein concentrate, authentication, smartphone video imaging, chemometrics, machine learning

## Abstract

With the growing interest in health and fitness, whey protein supplements are becoming increasingly popular among fitness enthusiasts and athletes. The surge in demand for whey protein supplements highlights the need for cost-effective methods to characterise product quality throughout the food supply chain. This study presents a rapid and low-cost method for authenticating sports whey protein supplements using smartphone video imaging (SVI) combined with machine learning. A gradient of colours ranging from purple to red is displayed on the front screen of a smartphone to illuminate the sample. The colour change on the sample surface is captured in a short video by the front-facing camera. Then, the video is split into frames, decomposed into RGB colour channels, and converted into spectral data. The relationship between video data and sample labels is established using machine learning models. The proposed method is tested on five tasks, including identifying 15 brands of whey protein concentrate (WPC), quantifying fat content and energy levels, detecting three types of adulterants, and quantifying adulterant levels. Moreover, the performance of SVI was compared to that of hyperspectral imaging (HSI), which has an equipment cost of around 80 times that of SVI. The proposed method achieves accuracies of 0.933 and 0.96 in WPC brand identification and adulterant detection, respectively, which are only around 0.05 lower than those of HSI. It obtains coefficients of determination of 0.897, 0.906 and 0.963 for the quantification of fat content, energy levels and milk powder adulteration, respectively. Such results demonstrate that the combination of smartphones and machine learning offers a low-cost and viable preliminary screening tool for verifying the authenticity of whey protein supplements.

## 1. Introduction

Whey protein supplements, derived from the by-products of cheese or curd production, are becoming increasingly popular among fitness enthusiasts. These supplements are rich in essential amino acids and are very effective in promoting muscle recovery and growth [1]. However, food fraud related to whey protein supplements, such as inconsistency between labelling and actual content, and adulteration with nitrogen-based substances, has caused great concern among consumers, retailers, and manufacturers [2]. Inferior and adulterated whey protein supplements not only have low nutritional value but are also more likely to trigger allergies, posing significant health risks and economic losses to consumers. Such substandard products also disrupt the market order and cause economic and reputational losses to honest businesses. As the current standard techniques for detecting whey protein supplements are often laboratory-based, they are difficult to implement throughout the supply chain [3,4]. Therefore, there is an urgent need to develop and apply new techniques that can assess the authenticity of whey protein supplements quickly and cost-effectively.

Traditionally, consumers have relied on their five senses to analyse the sensory attributes of whey protein supplements, such as colour, smell, texture, taste and solubility, in order to detect potential adulteration and verify product authenticity. However, such sensory analyses are usually subjective, inconsistent, unpredictable and destructive [5]. Standard chemical analysis is also available, including the Kjeldahl and Dumas methods for protein content determination, as well as high-performance liquid chromatography, ion-exchange chromatography and electrophoresis for the detection of non-bovine proteins and nitrogen-based adulterants [6,7,8]. However, despite offering high precision and excellent detection limits and reliability, these methods are not suitable for in situ, real-time and large-scale applications due to drawbacks, such as being time-consuming, having high costs and the need for specialised operational skills [9,10].

Recently, spectroscopic techniques such as Fourier-transform infrared (FTIR) spectroscopy and near-infrared (NIR) spectroscopy have been used to assess the authenticity and potential adulteration of whey protein supplements [11,12]. These techniques have the advantage of fast, non-destructive analysis and do not require complex sample preparation [13,14]. For instance, Andrade et al. utilised FTIR spectroscopy combined with chemometrics to effectively detect adulteration in whey protein concentrate (WPC) and accurately predicted the protein content and the amount of whey powder added [15]. Similarly, Martins et al. tested FTIR spectroscopy to detect and quantify wheat flour adulteration in WPC and showed that the method accurately predicted the degree of adulteration [7]. Nobari Moghaddam et al. and Lukacs et al. demonstrated that the use of NIR spectroscopy and chemometrics can efficiently identify and quantify common adulterants (milk powder and maltodextrin) and nitrogen-based adulterants [6,11]. Additionally, Raman spectroscopy and fluorescence spectroscopy have been used to detect and quantify adulterants in WPC, including creatine, glutamine, taurine, caffeine and lactose [2,16]. Compared to traditional analytical techniques, spectroscopic methods have significantly enhanced the efficiency and simplicity of verifying the authenticity of WPC. In particular, the use of miniaturised and portable spectrometers has dramatically reduced the costs and operational complexity involved in such assessments [1,17,18]. However, the high cost of spectrometers remains a barrier to their widespread adoption.

In a previous study, we developed a low-cost smartphone-based system for food authentication, namely smartphone video imaging (SVI) [19]. It generates a series of colours ranging from purple to red on the smartphone screen to illuminate the sample surface. The front-facing camera captures this process as short videos. To convert the obtained videos into data, the videos are split into frames and decomposed into RGB colour space, and then the region of interest (ROI) is selected. SVI has demonstrated its feasibility in quantifying adulterants in specific food products, such as assessing the levels of rapeseed oil in extra-virgin olive oil, turmeric powder in saffron powder, and minced pork in minced beef [20,21,22]. Moreover, it has also been used to verify the authenticity of products, for example, to distinguish between different types of ginseng, and to distinguish between whole milk, semi-skimmed milk and skimmed milk [19,23]. SVI is capable of capturing spectral and spatial data about samples within the visible range, exhibiting characteristics similar to those of hyperspectral imaging (HSI). When integrated with machine learning methods, it facilitates the establishment of a relationship between the video data and the analyte labels [24]. The performance of SVI has been verified to be superior to computer vision systems and comparable to HSI [19,21]. As a low-cost and user-friendly primary screening tool, SVI provides a potential solution for large-scale real-time assessment of the authenticity of whey protein supplements. This would provide consumers, suppliers, manufacturers and regulators with a convenient way to ensure product integrity and quality.

In this work, we present the use of SVI and machine learning methods to evaluate product authenticity and detect adulteration in whey protein supplements. The proposed approach is first utilised to differentiate 15 commercial WPC brands (Task 1) and to quantify the fat content (Task 2) and energy levels (Task 3). Three types of adulterants (maltodextrin, flour and milk powder) are then detected (Task 4), and the levels of milk powder adulteration are quantified (Task 5). Short videos of 345 WPC samples are recorded using a smartphone and converted into spectral-like data. Quantitative and qualitative relationships between video data and sample labels are established using machine learning and chemometrics models. In addition, the performance of SVI is evaluated and compared with that of HSI on the same tasks. Experimental results will demonstrate the feasibility of smartphone combined with machine learning for rapid and low-cost authentication of whey protein supplements.

## 2. Materials and Methods

### 2.1. Sample Preparation

**Figure 1:**
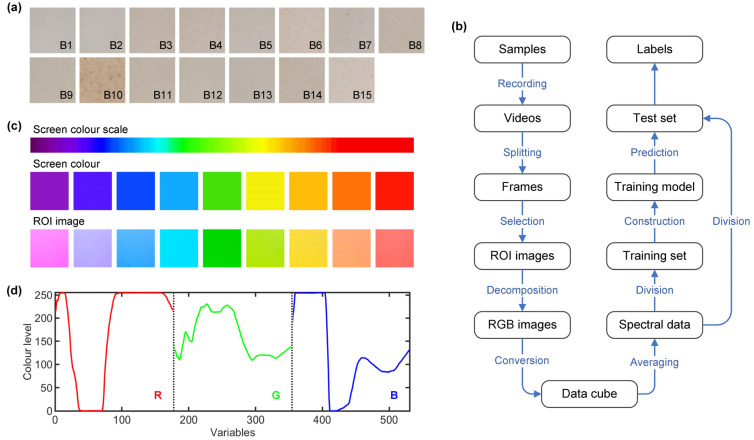
(**a**) Representative images of WPC samples; (**b**) flowchart from video acquisition to data analysis; (**c**) some ROI images and their corresponding screen colours; (**d**) video data of one sample.

A total of 15 brands of WPC products, labelled from B1 to B15, were acquired from various Chinese online retailers in 2024. The protein, carbohydrate and fat content (g/100 g) and energy levels (kJ/100 g) of these products, ranging from 15.7 to 85.7, 4.6 to 76.8, 0.013 to 0.101 and 1607 to 1776, respectively, were analysed in accordance with the Chinese national standard methods (GB 5009.5-2016 and GB/Z 21922-2008) [25,26]. The certified fat contents and energy levels of 15 brands of WPC are given in Table 1. For each brand, 12 samples, each weighing 2 g, were pressed at a pressure of 20 MPa to form compact and smooth pellets to improve signal quality and measurement repeatability. The pellets had a diameter of 30 mm and a thickness of 2.5 mm. Figure 1a depicts the surface images of WPC samples from various brands. Most of these samples were predominantly white, with some showing a more pronounced yellowish colour. Maltodextrins, wheat flour and milk powder were mixed separately in B1 WPC to prepare adulterated samples. The protein content (g/100 g) of these adulterants was not higher than 10, whereas that of B1 WPC was 85.7. The adulterant concentrations were varied from 5% to 50% (*w*/*w*) at 5% intervals, and 5 pellet samples were prepared for each adulteration level. The sample sizes for the five tasks were 180, 180, 180, 150 and 55, respectively.

### 2.2. Measurements

Figure 1b shows the process of video recording, image processing and data analysis. A series of gradient colours ranging from purple to red were generated on the screen of a smartphone (Samsung Galaxy S21+), as shown by the colour bar in Figure 1c. The smartphone screen was used to illuminate the sample and was placed parallel to the sample surface at a fixed distance of 10 cm. The front camera of the smartphone (10 megapixels, f/2.2 aperture) recorded the colour change process on the sample surface in a 5 s video. To avoid interference from inconsistent ambient light, all videos were recorded in a dark chamber and stored in MP4 format, with each file sized approximately 7 MB. The video was divided into 177 to 179 frames, each with a resolution of 960 × 720 pixels. To ensure a consistent number of frames, only the first 177 frames were selected. ROI images were then automatically cropped on the frames, with each image measuring 120 × 120 pixels and saved in JPG format, as depicted in Figure 1c. For each sample, these ROI images were decomposed into RGB colour channels and concatenated into a data cube. The average colour level for each image was calculated to form spectrum-like data, as shown in Figure 1d. The data matrix had 531 variables with colour levels ranging from 0 (dark) to 255 (light). The SVI application installed on Android systems is available at https://github.com/song29057/smartphone-video-imaging (accessed on 27 March 2025).

A benchtop HSI system (HSI-VNIR-B1621, Isuzu Optics) was used to obtain the reflectance spectra of WPC samples. This system, which operated in the visible-NIR region, comprised a spectrograph, an electron-magnifying charge-coupled device camera, a C-mount lens, two 150 W tungsten halogen lamps and a mobile sample stage [18]. During the scanning process, samples were placed on the mobile stage, which moved at a speed of 3.5 mm/s. The system performed line-by-line scans with an exposure time of 34 ms. The camera was located 15 cm above the samples, and the illumination intensity was set to 200 units. Hyperspectral images were captured at 342–1002 nm wavelengths (616 wavebands) with an 816-pixel spatial resolution per scan line. The corresponding pixel resolutions were approximately 1 nm spectrally and 0.07 mm spatially. The acquisition of hyperspectral images was conducted in a dark chamber. For each sample, an ROI measuring 100 × 100 pixels was selected, and the spectral data within this ROI were averaged.

### 2.3. Data Analysis

The data analysis process comprises three key stages: sample division, model construction and label prediction, as shown in Figure 1b. Samples were first randomly divided into training and test samples in a ratio of 2:1. The training samples were used to build and optimise the model, while the labels of the test samples were predicted by the model and compared with the actual labels. The video data and HSI data were normalised to the [0, 1] range to reduce scaling differences and improve model performance. To visualise the class separability, the data were projected into low-dimensional space using principal component analysis (PCA). PCA uses orthogonal transformations to convert a set of potentially correlated variables into a new set of linearly uncorrelated variables. The PCA score plot graphically represents the sample distribution in the reduced-dimensional space defined by the principal components.

Further, the relationship between the spectral data and corresponding sample labels was established using chemometrics and machine learning models. Partial least squares regression (PLSR) is one of the most frequently used chemometrics methods for addressing high-dimensionality and multicollinearity problems [27]. PLSR identifies latent variables that maximise the covariance between predictors and responses. With these latent variables, a linear model can be constructed to relate predictors to responses. To improve the prediction performance, the PLSR model was optimised by selecting the optimal number of latent variables. PLSR may suffer from performance degradation when dealing with data with a high degree of nonlinearity [28,29]. Partial least squares discriminant analysis (PLS-DA) is a variant of PLSR designed for classification tasks. It transforms class labels into a dummy matrix to handle categorical responses [30]. Kernel extreme learning machine (K-ELM) extends the traditional extreme learning machine (ELM) by incorporating a nonlinear kernel function to enhance its capability to handle complex and nonlinear data [31]. Unlike ELM, which uses random input weights and least squares solutions for output weights in a single hidden layer feedforward network, K-ELM maps input data into a high-dimensional space through kernel functions. K-ELM has been commonly used to analyse spectral data due to its fast calculation speed and good generalisation ability [32,33].

The parameters for machine learning models were optimised through 10-fold cross-validation on the training data. For PLSR and PLS-DA, the optimal number of latent variables was selected within the range of 1 to 10. In the case of K-ELM, which employs a radial basis function (RBF) kernel, the regularisation and kernel parameters were searched across a logarithmic scale from 2^1^ to 2^10^. The classification result was primarily evaluated based on accuracy. The quantification performance was assessed by calculating the root mean square error (RMSE), mean absolute error (MAE) and coefficient of determination (R^2^) during the training and test phases. The image processing and data analysis were implemented in MATLAB R2024a. The experimental data and analysis codes are available at https://github.com/song29057/SVI-WPC-detection-2025 (accessed on 1 April 2025).

## 3. Results and Discussion

### 3.1. Data Description

**Figure 2:**
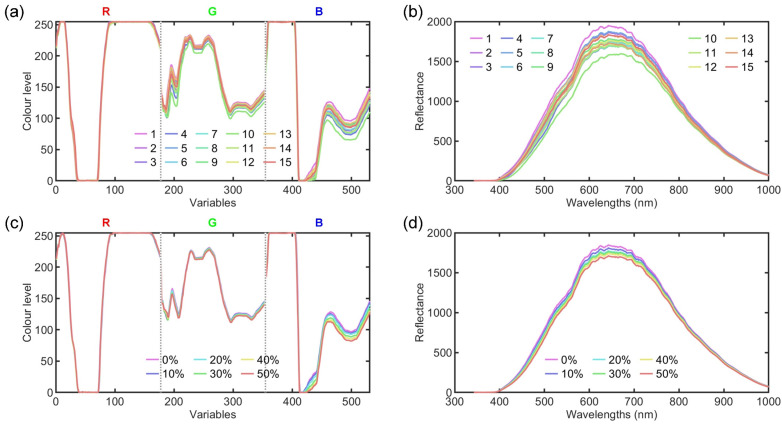
Average video (**a**) and HSI (**b**) data for WPC of various brands, and average video (**c**) and HSI (**d**) data for WPC adulterated with varying milk powder concentrations.

Figure 2a,b show the average video and HSI data for the 15 WPC brands. The video data exhibit notable fluctuations on all three RGB channels, with some variables reaching extreme values of 0 and 255. Class separability is evident in the green channel (variables 178–354) and specific parts of the blue channel (variables 450–531), while there is considerable overlap between the different classes throughout the red channel and parts of the blue channel. The raw HSI data show an increase in reflectance from 0 as the wavelength increases, peaking around 650 nm with peak values ranging from 1500 to 2000. Subsequently, the reflectance values decrease to about 70 as the wavelength increases. Although all samples appear predominantly white, those with slightly darker surface tones exhibit marginally lower reflectance values. Generally, the HSI data show a clear class separability over the 500–750 nm range. Figure 2c,d present the average video and HSI data for WPC adulterated with varying levels of milk powder. Differences in the level of adulteration are evident in the video data within the variable range of 420–531, and the HSI data within the range of 500–750 nm.

### 3.2. WPC Authentication

**Figure 3:**
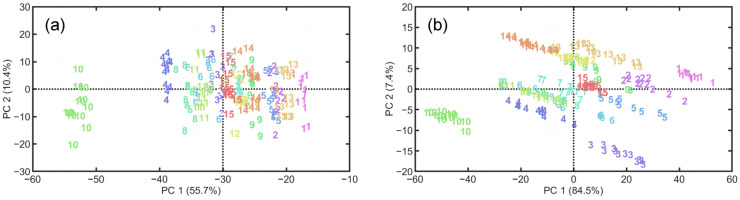
PCA projection of video data (**a**) and HSI data (**b**) for 15 commercial WPC brands in two-dimensional space.

Figure 3 presents the low-dimensional projection of the pre-processed video and HSI data using PCA, demonstrating the class separability of WPC samples. For the projected video data, the first two principal components (PCs) explain 55.7% and 10.4% of the variation, respectively. Along the *x*-axis, data points belonging to different classes exhibit a certain degree of separability. While the mapped HSI data demonstrate better class separability, with most data points clustered in different regions. The first two PCs explain more than 91% of the variation, suggesting a certain degree of multicollinearity in the HSI data. Further, machine learning methods are employed to establish relationships between the pre-processed data and sample labels for WPC brand classification. In the training phase, PLS-DA and K-ELM achieve accuracies of 0.658 and 0.883, respectively, when using video data. With HSI data, the accuracies are 0.992 and 1 for the two methods, respectively. In the testing phase, PLS-DA and K-ELM achieve accuracies of 0.73 and 0.933, respectively, using video data. Using K-ELM, two samples from B11 are misclassified as B8, and two samples from brand B12 are misclassified as B7 and B9, respectively. For HSI data, the accuracies are 0.95 for PLS-DA and 0.983 for K-ELM. Multiple classes coupled with low-quality video data significantly reduce the accuracy of PLS-DA. In contrast, K-ELM, due to its higher model complexity, can effectively capture features in nonlinear data, thus significantly improving the classification accuracy. These results suggest that SVI combined with machine learning can effectively differentiate WPC brands, with performance comparable to that of HSI.

**Figure 4:**
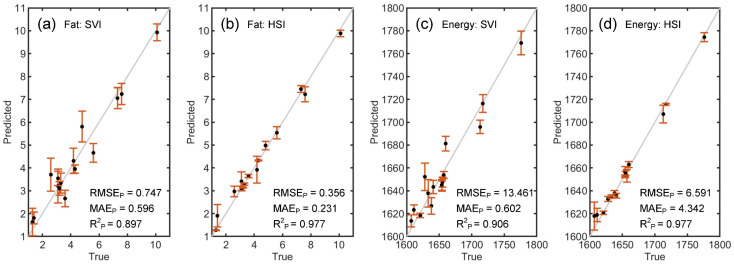
True versus predicted values of fat contents (g/100 g) quantification based on SVI (**a**) and HSI (**b**) and those of energy levels (kJ/100 g) quantification based on SVI (**c**) and HSI (**d**) combined with K-ELM.

Table 2 shows the results of quantifying fat content (g/100 g) and energy levels (kJ/100 g) of WPC using SVI and HSI coupled with machine learning models. The subscripts cv and p denote cross-validation and prediction, respectively. It is worth noting that the results obtained by K-ELM are significantly better than those obtained by PLSR. For fat content quantification, the R^2^_CV_ values for both instruments are above 0.92 in most cases during the training phase. In the testing phase, the highest R^2^_P_ values based on SVI and HSI are 0.897 and 0.977, respectively. The corresponding RMSE_P_ values are 0.747 and 0.356, respectively. For energy level quantification, the R^2^_CV_ values range from 0.881 to 0.982. The highest R^2^_P_ values based on SVI and HSI are 0.906 and 0.977, respectively. The corresponding RMSE_P_ values are 13.46 and 6.591, respectively. Figure 4 illustrates the true versus predicted fat contents based on SVI and HSI combined with K-ELM, where the predicted values closely align with the certified values to a large extent. These results demonstrate the feasibility of combining SVI with machine learning in quantifying fat content and energy levels of WPC.

**Figure 5:**
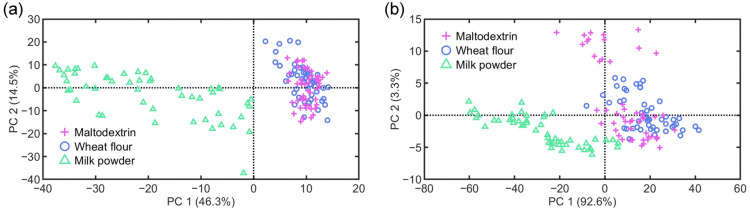
PCA projection of video data (**a**) and HSI data (**b**) for samples with three adulteration types in two-dimensional space.

Figure 5 shows the projection of video and HSI data for three types of adulterated samples into the low-dimensional space using PCA. The milk powder adulterant in WPC exhibits good separability from other types of adulterants. Based on the unsupervised dimensionality reduction, it is difficult to distinguish between maltodextrin and wheat flour adulterants in WPC. In the training phase, PLS-DA and K-ELM achieve classification accuracies of 0.93 and 0.95, respectively, using video data. When using HSI data, these classifiers achieve accuracies of 1 and 0.96, respectively. In the testing phase, for video data, PLS-DA misclassifies 2 out of 50 samples, while K-ELM misclassifies 3 out of 50 samples. For HSI data, the two classifiers achieve perfect classification accuracy. These results indicate that the three distinct adulterants can be effectively distinguished based on SVI measurements.

**Figure 6:**
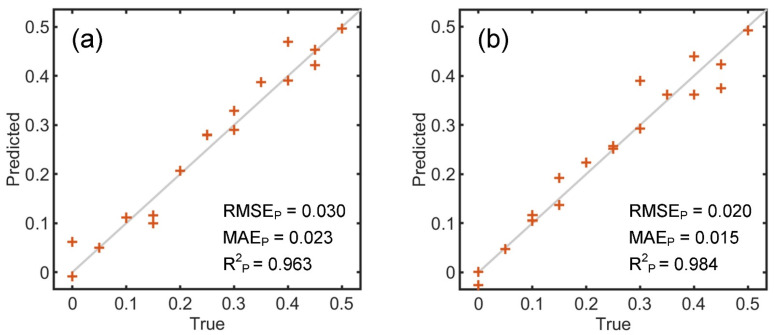
True versus predicted levels of milk powder adulteration in WPC based on SVI (**a**) and HSI (**b**) combined with K-ELM (18 test samples). Due to image scaling, in the left figure, the cross markers for the 0.1 true level overlap, and the cross markers for the 0.25 true level overlap.

Table 3 provides a comparison of SVI and HSI measurements in quantifying milk powder adulteration in WPC. Based on the video data, the PLSR and K-ELM models achieve R^2^_P_ values of 0.959 and 0.963, respectively, on the test dataset. The corresponding RMSE_P_ and MAE_P_ are not higher than 0.032 and 0.024, respectively. When using HSI data, the R^2^_P_ values for PLSR and K-ELM are 0.951 and 0.984, respectively. K-ELM yields the lowest RMSE_P_ and MAE_P_ of 0.02 and 0.015, respectively. Figure 6 shows the true and predicted levels of adulteration based on SVI and HSI combined with K-ELM. The predicted values exhibit a strong correspondence with the true values, suggesting that the two imaging techniques can offer viable tools for quantifying milk powder adulteration in WPC.

### 3.3. Discussion

The results demonstrate that SVI combined with machine learning can effectively address the five tasks specified in the Introduction: classifying different brands of WPC, quantifying fat content and energy levels, detecting three common types of adulteration, and quantifying milk powder adulteration in WPC. Due to the relatively small sample sizes (<200), this study uses simple machine learning method for data analysis. These methods maintain lower model complexity, thereby reducing potential overfitting risks. Specifically, classification models achieve accuracies of 0.933 for WPC brand classification and 0.96 for adulterant detection based on video data, which are approximately 0.05 lower than those obtained using HSI data. For the quantification of fat content, energy levels, and milk powder adulteration, SVI combined with K-ELM yields R^2^_P_ values of 0.897, 0.906 and 0.963, respectively. In comparison, HSI combined with K-ELM achieves higher R^2^_P_ values of 0.977, 0.977 and 0.984, respectively. Although the performance of SVI in WPC authentication is lower than that of HSI, its hardware cost is only 1/80 of the benchtop HSI used in this study. Furthermore, the portability of SVI makes it more suitable for on-site and real-time WPC authentication. However, the applicability of SVI is limited to specific tasks due to the challenge of directly correlating colour characteristics with chemical composition and content. Additionally, the generalisability of models trained on video data from one smartphone across different devices remains to be investigated, potentially through device calibration and transfer learning to address this issue.

## 4. Conclusions

This study combines SVI with machine learning to identify different brands of WPC, quantify fat content and energy levels, detect three types of adulterants, and quantify milk powder adulteration in WPC. The colour levels of 15 brands of WPC and adulterated WPC samples were captured under varying coloured light illuminations using smartphone video recordings. Classification and regression methods, including PLS-DA, PLSR and K-ELM, were used to extract features from the video data and establish relationships between the video data and sample labels. The performance of SVI was evaluated by comparing it with a benchtop HSI system. The results demonstrate that SVI, when combined with machine learning, can provide a viable and cost-effective solution for WPC authentication. The advantages of simple operation, low cost, portability, and fast measurement make SVI an attractive solution for the authentication of whey protein supplements, providing a practical option for consumers, suppliers, manufacturers and regulatory agencies. Our future work will explore combining SVI with spectroscopic techniques to quantify protein and carbohydrate contents in WPC. In addition, the use of SVI and machine learning for the authentication of other dairy products will also be investigated.

## Figures and Tables

**Table 1:** The certified fat contents and energy levels of 15 commercial WPC brands.

	B1	B2	B3	B4	B5	B6	B7	B8	B9	B10	B11	B12	B13	B14	B15
Fat (g/100 g)	3.3	3.1	4.3	7.3	3.2	3.1	5.6	7.6	3.6	10.1	4.8	2.6	1.4	4.2	1.3
Energy (kJ/100 g)	1657	1633	1655	1717	1654	1607	1654	1713	1638	1776	1660	1628	1611	1641	1621

**Table 2:** Performance of SVI and HSI for quantifying fat content (g/100 g) and energy levels (kJ/100 g) of WPC. The subscripts cv and p denote cross-validation and prediction, respectively.

Analyte	Measurement	Model	RMSE_CV_	MAE_CV_	R^2^_CV_	RMSE_P_	MAE_P_	R^2^_P_
Fat	SVI	PLSR	0.774	0.628	0.889	0.916	0.760	0.845
(g/100 g)		K-ELM	0.633	0.535	0.926	0.747	0.596	0.897
	HSI	PLSR	0.569	0.463	0.940	0.589	0.482	0.936
		K-ELM	0.298	0.218	0.984	0.356	0.231	0.977
Energy	SVI	PLSR	15.111	12.415	0.881	17.567	14.128	0.840
(kJ/100 g)		K-ELM	12.769	10.691	0.915	13.460	10.602	0.906
	HSI	PLSR	12.661	10.350	0.917	13.390	11.094	0.907
		K-ELM	5.887	4.185	0.982	6.591	4.342	0.977

**Table 3:** Performance of SVI and HSI for quantifying milk powder adulteration in WPC.

		RMSE_CV_	MAE_CV_	R^2^_CV_	RMSE_P_	MAE_P_	R^2^_P_
SVI	PLSR	0.039	0.031	0.940	0.032	0.024	0.959
	K-ELM	0.040	0.032	0.937	0.030	0.023	0.963
HSI	PLSR	0.026	0.018	0.973	0.034	0.024	0.951
	K-ELM	0.030	0.023	0.963	0.020	0.015	0.984

## Data Availability

The original contributions presented in the study are included in the article, further inquiries can be directed to the corresponding authors.

## References

[1] Wang T., Tan S.Y., Mutilangi W., Aykas D.P., Rodriguez-Saona L.E. (2015). Authentication of Whey Protein Powders by Portable Mid-Infrared Spectrometers Combined with Pattern Recognition Analysis. J. Food Sci..

[2] Pereira C.G., Andrade J., Ranquine T., de Moura I.N., da Rocha R.A., Furtado M.A.M., Bell M.J.V., Anjos V. (2018). Characterization and Detection of Adulterated Whey Protein Supplements Using Stationary and Time-Resolved Fluorescence Spectroscopy. LWT.

[3] Wang K., Sun D.-W., Pu H., Wei Q. (2017). Principles and Applications of Spectroscopic Techniques for Evaluating Food Protein Conformational Changes: A Review. Trends Food Sci. Technol..

[4] Di Rosa A.R., Leone F., Cheli F., Chiofalo V. (2017). Fusion of Electronic Nose, Electronic Tongue and Computer Vision for Animal Source Food Authentication and Quality Assessment—A Review. J. Food Eng..

[5] Özdoğan G., Lin X., Sun D.-W. (2021). Rapid and Noninvasive Sensory Analyses of Food Products by Hyperspectral Imaging: Recent Application Developments. Trends Food Sci. Technol..

[6] Lukacs M., Bazar G., Pollner B., Henn R., Kirchler C.G., Huck C.W., Kovacs Z. (2018). Near Infrared Spectroscopy as an Alternative Quick Method for Simultaneous Detection of Multiple Adulterants in Whey Protein-Based Sports Supplement. Food Control.

[7] Martins M.S., Nascimento M.H., Barbosa L.L., Campos L.C.G., Singh M.N., Martin F.L., Romão W., Filgueiras P.R., Barauna V.G. (2022). Detection and Quantification Using ATR-FTIR Spectroscopy of Whey Protein Concentrate Adulteration with Wheat Flour. LWT.

[8] Saxton R., McDougal O.M. (2021). Whey Protein Powder Analysis by Mid-Infrared Spectroscopy. Foods.

[9] Fu X., Kim M.S., Chao K., Qin J., Lim J., Lee H., Garrido-Varo A., Pérez-Marín D., Ying Y. (2014). Detection of Melamine in Milk Powders Based on NIR Hyperspectral Imaging and Spectral Similarity Analyses. J. Food Eng..

[10] Su W.H., Sun D.W. (2018). Fourier Transform Infrared and Raman and Hyperspectral Imaging Techniques for Quality Determinations of Powdery Foods: A Review. Compr. Rev. Food Sci. Food Saf..

[11] Nobari Moghaddam H., Tamiji Z., Amini M., Khoshayand M.R., Kobarfrad F., Sadeghi N., Hajimahmoodi M. (2024). Development of Non-Destructive Methods for the Assessment of Authenticity of Sports Whey Protein Supplements. Food Addit. Contam. Part A.

[12] De Géa Neves M., Poppi R.J., Breitkreitz M.C. (2022). Authentication of Plant-Based Protein Powders and Classification of Adulterants as Whey, Soy Protein, and Wheat Using FT-NIR in Tandem with OC-PLS and PLS-DA Models. Food Control.

[13] Meenu M., Cai Q., Xu B. (2019). A Critical Review on Analytical Techniques to Detect Adulteration of Extra Virgin Olive Oil. Trends Food Sci. Technol..

[14] Danezis G.P., Tsagkaris A.S., Camin F., Brusic V., Georgiou C.A. (2016). Food Authentication: Techniques, Trends & Emerging Approaches. TrAC-Trends Anal. Chem..

[15] Andrade J., Pereira C.G., de Almeida Junior J.C., Viana C.C.R., de Oliveira Neves L.N., da Silva P.H.F., Bell M.J.V., dos Anjos V.d.C. (2019). FTIR-ATR Determination of Protein Content to Evaluate Whey Protein Concentrate Adulteration. LWT.

[16] Jiao X., Meng Y., Wang K., Huang W., Li N., Liu T.C.-Y. (2019). Rapid Detection of Adulterants in Whey Protein Supplement by Raman Spectroscopy Combined with Multivariate Analysis. Molecules.

[17] Zinia Zaukuu J.-L., Aouadi B., Lukács M., Bodor Z., Vitális F., Gillay B., Gillay Z., Friedrich L., Kovacs Z. (2020). Detecting Low Concentrations of Nitrogen-Based Adulterants in Whey Protein Powder Using Benchtop and Handheld NIR Spectrometers and the Feasibility of Scanning through Plastic Bag. Molecules.

[18] Song W., Yun Y., Lv Y., Zhang C., Tang X., Wang H., Wang Z. (2025). Authentication and Quality Assessment of Whey Protein-Based Sports Supplements Using Portable near-Infrared Spectroscopy and Hyperspectral Imaging. Food Res. Int..

[19] Song W., Wang H., Yun Y. (2025). Smartphone Video Imaging: A Versatile, Low-Cost Technology for Food Authentication. Food Chem..

[20] Song W., Song Z., Vincent J., Wang H., Wang Z. (2020). Quantification of Extra Virgin Olive Oil Adulteration Using Smartphone Videos. Talanta.

[21] Song W., Wei X., Wang H., Xu J., Tang X., Kong X. (2024). Rapid and Low-Cost Detection of Saffron (*Crocus sativus* L.) Adulteration Using Smartphone Videos and Spectral Data Fusion Strategy. J. Food Compos. Anal..

[22] Song W., Yun Y., Wang H., Hou Z., Wang Z. (2021). Smartphone Detection of Minced Beef Adulteration. Microchem. J..

[23] Song W., Jiang N., Wang H., Vincent J. (2020). Use of Smartphone Videos and Pattern Recognition for Food Authentication. Sens. Actuators B Chem..

[24] Song W., Song Z., Yue X., Zhu Z., Wang J., Wang H., Wang Z. (2025). Siamese Network-Based Spectral Reconstruction for Rapid Identification of Fire-Retardant Coatings. Measurement.

[25] (2016). National Food Safety Standard: Determination of Protein in Foods.

[26] (2008). Fundamental Terminology and Definition of Nutritional Component in Foods.

[27] Mehmood T., Ahmed B. (2016). The Diversity in the Applications of Partial Least Squares: An Overview. J. Chemom..

[28] Song W., Afgan M.S., Yun Y., Wang H., Cui J., Gu W., Hou Z., Wang Z. (2022). Spectral Knowledge-Based Regression for Laser-Induced Breakdown Spectroscopy Quantitative Analysis. Expert Syst. Appl..

[29] Chen X., Zhong W., Jiang C., Li Z., Peng X., Cheng H. (2020). Key Performance Index Estimation Based on Ensemble Locally Weighted Partial Least Squares and Its Application on Industrial Nonlinear Processes. Chemom. Intell. Lab. Syst..

[30] Barker M., Rayens W. (2003). Partial Least Squares for Discrimination. J. Chemom..

[31] Huang G.-B., Zhu Q.-Y., Siew C.-K. (2006). Extreme Learning Machine: Theory and Applications. Neurocomputing.

[32] Ding Y., Yan F., Yang G., Chen H., Song Z. (2018). Quantitative Analysis of Sinters Using Laser-Induced Breakdown Spectroscopy (LIBS) Coupled with Kernel-Based Extreme Learning Machine (K-ELM). Anal. Methods.

[33] Zheng W., Shu H., Tang H., Zhang H. (2019). Spectra Data Classification with Kernel Extreme Learning Machine. Chemom. Intell. Lab. Syst..

